# Charting a path towards a public health approach for gambling harm prevention

**DOI:** 10.1007/s10389-020-01437-2

**Published:** 2021-01-07

**Authors:** Alex Price, Margo Hilbrecht, Rosa Billi

**Affiliations:** 1 Centre for the Advancement of Best Practices, The Responsible Gambling Council, 205-411 Richmond Street East, Toronto, Ontario M5A 3S5 Canada; 2 Gambling Research Exchange, 214A-55 Wyndham Street North, Guelph, Ontario N1H 7T8 Canada; 3 University of Waterloo, 200 University Ave W, Waterloo, Ontario N2L 3G1 Canada; 4 Victoria Responsible Gambling Foundation, Level 6, 14–20 Blackwood Street, North Melbourne, Victoria 3051 Australia

**Keywords:** Gambling harm, Public health model, Essential functions, Critical review, Health assessment, Surveillance

## Abstract

**Aim:**

Gambling harm is a serious public health issue affecting the health, financial security, and social well-being of millions of people and their close relations around the world. Despite its population health implications, gambling harm is not typically viewed and treated as a public health policy issue. This paper critically reviews the evolution of the public health perspective on gambling harm. It also considers how gambling harm can be operationalized within a public health model.

**Methods:**

A critical historical review of the emerging public health perspective on gambling harm was conducted. Key documents covering three decades of development were reviewed and appraised through a process of deliberation and debate over source impact in the fields of research, policy, and programming internationally.

**Results:**

The first decade mainly focused on identifying gambling harm and framing the public health issue. The second decade featured the expansion of health assessment and emerging areas of policy and program development. The third decade saw an increased focus on public health frameworks that advanced understanding of harm mechanics and impact. As reflected by the *essential functions* of a general public health model, gambling harm prevention efforts emphasize health promotion over other key functions like health assessment and surveillance.

**Conclusion:**

Gambling harm is a public health issue requiring greater attention to health assessment and surveillance data development.

## Introduction

The development of modern public health models began nearly 200 years ago. This health service paradigm has significantly shaped how we prevent, prepare for, and treat human health conditions at an individual and population level. Gambling-related harms, however, have not garnered the same level of attention from these health service models as other acute and chronic health conditions. Despite these circumstances, the resources, knowledge, and skill sets embedded in public health systems and models may offer substantial benefits for addressing gambling harms.

Gambling harms constitute a serious public health issue. Worldwide, an estimated 0.1% to 5.8% of adult populations experience serious problems with gambling (Calado and Griffiths 2016). Although the health, economic, social, and personal harms of gambling are most severe among problem gamblers, they can extend across the risk spectrum (Blaszczynski 2009; Browne et al. 2016; Langham et al. 2016). Further, the impact of these harms affects not only the gambler, but also radiates with negative implications for family, friends, workplaces, and communities (Langham et al. 2016). Though much of the research and policy interest in gambling has focused on prevalence rates and downstream treatment of people with acute gambling problems (Productivity Commission 2010), interest in harm prevention from a public health perspective is growing (Browne et al. 2016; Wardle et al. 2019).

This paper presents a critical review on the evolution of the public health perspective on gambling harms, at a high level. It also considers how a public health model can be operationalized to address gambling harms, though it acknowledges that differences across jurisdictions and local contexts can affect public health implementation efforts. Generally, the authors hope that this paper will prompt discussion and debate concerning how this approach can contribute to a growing evidence base and catalyze the development of policies attending to gambling-related harm at a population health level.

## Methods

A critical historical literature review of the public health perspective and approach to gambling harm prevention and minimization was conducted between December 2019 and September 2020. Forty-seven documents, both peer-reviewed and grey sources, covering three decades and representing distinct eras of knowledge and theoretical development were reviewed (Grant and Booth 2009). The literature search was conducted by three investigators who collectively have over 20 years in the field of gambling research and knowledge transfer and exchange, internationally. An iterative process of group deliberation and debate was used to appraise literature based on impact in the field of research, policy, and programming internationally (e.g., in Canada, Australia, New Zealand, Sweden, the United Kingdom, and other international jurisdictions). This process of deliberation and literature appraisal, which centred on the concepts of “gambling harm” and “public health approaches for gambling,” were facilitated through email, video conferencing, telephone discussions, and Google Docs collaborative functions for notation and commentary.

A critical analysis of harm prevention and minimization stemming from the historical review was also carried out, based upon a generalized framework of a modern public health system. This framework reflects developments beginning in the 1990s by the US Centers for Disease Control and Prevention (CDC) and the World Health Organization (WHO) on *essential public health functions* (World Health Organization 2018). A discussion on further integrating gambling harm prevention and minimization within a public health approach followed.

### Situating gambling harm and modern health service models

The nature and evolution of modern *public health* models provide an important reference point for understanding the potential benefits of an integrated focus on gambling-related harms.

#### The public health model

The modern public health model emerged in parallel with the biomedical model. In late nineteenth-century Europe, following the Industrial Revolution, public health issues and disease threats stemming from urbanization, industrial production, and the concentration of large populations in relatively small areas were apparent. Food- and water-borne illnesses, the potential for rapid outbreaks of infectious diseases, and other threats saw public health systems respond with a focus on sanitation, food and water safety, the development of vaccines and antibiotics, and the expansion of epidemiology and laboratory sciences (DeSalvo et al. 2017).

In the second half of the twentieth century, public health turned its focus to the growing burden of chronic disease and new emerging infectious diseases like HIV/AIDS. The evolution of this *new* public health model was in part characterized by an appreciation for the social determinants of health and the formalization of *essential functions* for public health systems (Bettcher et al. 1998; BCMHS 2005; DeSalvo et al. 2017).

While slight international variations exist, essential public health functions generally reflect the capacity for (1) *health promotion*, (2) *health protection*, (3) *disease and injury prevention*, (4) *population health assessment*, and (5) *health surveillance*. A key feature and strength of this model, which persists to this day, is the ability to operationalize and manage complex population health issues and their social determinants. Rigorous data collection, analysis, and informed decision-making have been central factors enabling public health systems to measure performance, set targets, and pursue population health improvement (Martin-Moreno et al. 2016).

#### Gambling harm and public health

Gambling is a globally prevalent activity with social and cultural value that also carries with it inherent public health risks (Raylu and Oei 2004; Banks 2017). Gambling harms can take the form of financial insecurity, employment disruption, suicide, substance abuse, psychological disorders, and more (Li et al. 2017). Importantly, gambling harms can have distributed effects extending beyond the individual to include interpersonal, community, and societal levels of impact (Li et al. 2017). However, much like harms from tobacco and alcohol abuse, negative health outcomes stemming from gambling have historically been viewed as individualized problems to be treated clinically, reflecting a biomedical disease model (Herzberg 2002; Potenza and Hollander 2002; Campbell and Smith 2003).

In contrast, alcohol and tobacco are now commonly viewed as population and public health issues supported by strategies, policies, and interventions to reduce downstream harms through upstream prevention efforts (De Beyer and Brigden 2004; Butler et al. 2017). Contemporary public health approaches to mitigate alcohol and tobacco harms include and extend from individual users and consider multiple social determinants of health (Thornton et al. 2016; Artiga and Hinton 2018). For instance, taxation, marketing regulations, restrictions on access and availability, community health services, and health promotion have had a substantial impact on population health outcomes (Burton et al. 2017; García-Esquinas et al. 2018).

While some elements of the public health model have seen adoption in the gambling field, particularly health promotion as reflected in *responsible gambling* initiatives, most efforts do not reflect a robust and coordinated public health approach (Gambling Commission 2019).

## Findings

### Gambling harm: the evolution of a public health perspective

Over the past three decades, research and policy initiatives have demonstrated the novelty of the public health approach to addressing gambling-related harm. Distinguishing between *gambling harm* and *problem gambling* has been an important step in conceptualizing a population approach to prevention and harm minimization (Browne and Rockloff 2017).

### The first decade: early initiatives

The first, formative decade of development in the 1990s focused mainly on identifying gambling harm and framing it as a public health issue. Early foundational research established a prism for understanding key factors from a social determinants of health perspective.

Harm as a public health issue first began appearing in the literature following the 1990 New Zealand National Survey of Problem and Pathological Gambling. During the second phase of the survey, respondents were asked about the harms and benefits of gambling related to personal pleasures and costs, relationships, employment, financial impacts, and legal issues (Abbott and Volberg 1992, 1996). At the population level, gender, age, ethnicity, employment status, having a family history of gambling, and participating regularly in continuous forms of gambling were identified as key risk factors. The authors also noted that as pathological gambling gained more acceptance as a significant public health problem, governments and health professionals would be under pressure to develop effective responses to gambling expansion. In 1997, New Zealand’s Ministry of Health published a report on population mental health with a chapter on problem gambling that supported a public health approach to harm prevention (Chetwynd 1997). A year later in Canada, the issue of gambling as a public health issue was also addressed in a report to the municipal councils in what is now the Region of Peel, Ontario (Cole 1998).

The first Australian survey of gambling-related problems was conducted in 1991 and shared the same measures used in the 1990 New Zealand study (Dickerson et al. 1996). Among people with gambling problems, negative impacts emerged in the forms of personal and interpersonal distress, employment disruption, and financial and legal issues. McMillen (1997) emphasized the importance of redefining gambling harm through a public health lens. She argued for a proactive approach that would highlight social structural factors like the policies and practices of government and industry, environmental factors such as game design, and the role of socio-cultural and material circumstances.

As early as 1994 in the United States, Volberg argued that as the number of venues increased, policymakers must address the unequal distribution of harm across various demographic groups, including at-risk groups such as women, children, and minorities (Volberg 1994). A few years later, in 1998, the first comprehensive review of comorbidity studies indicated the high level of co-occurrence with substance use, personality, and mood disorders. The authors called for more research taking age, sex, and ethnicity into consideration, recognizing the potential for different demographic impacts of gambling harms (Crockford and el-Guebaly 1998). When considered together, these findings raised questions of a need for a multi-sectoral approach to addressing gambling harm at the population level.

By 1999, Korn and Shaffer’s *Gambling and Health Framework* (Korn and Shaffer 1999) presented an integrated view of findings from the past decade and linked them to public health strategies such as the WHO Ottawa Charter for Health Promotion. This strategy took note of important individual, community, and institutional levels of health impact in order to guide efforts to improve well-being. Specifically, Korn and Shaffer’s framework supported a public policy orientation aimed at harm prevention and reduction; it considered both the benefits and detriments of gambling, it promoted balanced choices, and it recognized the need to protect vulnerable populations (World Health Organization 1986). Moreover, it argued that all levels of gambling across the risk spectrum should be included.

### The second decade: research and policy advances

During the second decade, there was progress in expanding health assessment and identifying policy- and program-relevant areas of development. In the early 2000s, a report commissioned by the Problem Gambling Committee of New Zealand recognized the importance of addressing the continuum of gambling harm as well as the complexity of contributing factors. The report argued that a variety of approaches should be adopted to accommodate different population groups, gambling types, and gambling environments. Health was recommended as foremost among government departments to develop a policy that would include treatment, harm minimization, and health promotion (Brown and Raeburn 2001). In 2005, the Australian Ministerial Council on Gambling published a report that grappled with definitions of problem gambling and harm in order to advance a gambling research agenda to support policy initiatives (Neal et al. 2005). It drew upon an extensive review of evidence in the academic and grey literature along with broad stakeholder contributions. The result was a nuanced collection of perspectives on problem gambling and harm that highlighted the strengths and weaknesses of individual- and population-focused approaches. Relevant to the public health model, the report recognized the importance of gambling-related harm as a community health issue.

Public health research on gambling harm progressed slowly during this decade, with some exceptional developments helping to advance the field of policy and research. For instance, New Zealand passed the Gambling Act in 2003, officially recognizing gambling as a public health issue. The act affirmed that all strategies regarding problem gambling must include harm minimization and prevention, public health promotion, treatment services for problem gamblers and their families, independent gambling-related research, and evaluation (Government of New Zealand 2003). Although not without industry criticism (Adams and Rossen 2012), it signalled that a public health approach could be adopted in government policy to address harm at the population level. In other parts of the world, evidence also began to emerge supporting a public health perspective on gambling. In Canada, for example, researchers embarked on an initiative exploring low-risk gambling thresholds based on gambling frequency and expenditure—this work will soon culminate in the world’s first set of low-risk gambling guidelines for the public (Currie et al. 2006, 2009).

Policy suggestions for adopting a public health paradigm were advanced in other countries, although not formally adopted. In the United States, Shaffer (2003) offered four principles for public health as a framework for gambling: scientific evidence base as the foundation of public health knowledge, public health knowledge derived from population-level research, proactive health initiatives that prioritize health promotion and prevention before treatment, and a balanced consideration of both the benefits and costs of gambling. In Canada, complementary underlying principles of a public health paradigm for gambling policy included the prevention of gambling-related problems among individuals and at-risk groups, the promotion of balanced and informed attitudes and behaviours by individuals and communities, and the protection of vulnerable groups at higher risk of gambling-related harm (Korn et al. 2003). Both sets of recommended principles recognize the importance of an evidence base that extends beyond individuals with gambling problems to include the full risk spectrum.

This decade also saw the development of four population-level longitudinal cohort studies exploring the relationships between gambling risk factors and health outcomes. Two studies were conducted in Canada: the Leisure, Lifestyle and Lifecycle Project (LLLP), undertaken by the Alberta Gambling Research Institute and situated in four urban centres in Alberta (el-Guebaly et al. 2015), and the Quinte Longitudinal Study (QLS), funded by the Ontario Problem Gambling Research Centre and based in the Quinte region of Ontario (Williams et al. 2015). Each included four waves of data collection across a 6-year period from 2006 to 2011. The Swedish Longitudinal Gambling Study (Swelogs), funded by the Public Health Agency of Sweden, began 2 years later in 2008. It included four waves of data collection, with the final telephone interviews taking place in 2013 (Abbott et al. 2017b; Romild et al. 2014). The Victorian Gambling Study (VGS), supported by the Victorian Department of Justice, also launched in 2008, with the fourth and final wave of data collection in 2011 (Victoria Department of Justice 2009; Victorian Responsible Gambling Foundation 2011). Subsequent longitudinal cohort studies conducted in the decade that followed included the New Zealand National Gambling Study (2012 to 2015), funded by the New Zealand Ministry of Health (Abbott et al. 2017a), and the Massachusetts Gambling Impact Cohort Study (MAGIC), supported by the Massachusetts Gaming Commission, the state regulatory agency. It began in 2015 and is ongoing (Mazar et al. 2019).

Collectively, these studies confirmed that there was considerable movement in transitions into and out of problem gambling. Between one-half to two-thirds of people in the high-risk category were problem gamblers who had relapsed. Along with validated measures of problem gambling risk and prevalence, the longitudinal studies included a full range of demographics that allowed insights into the role of various social determinants of health. This body of evidence also drew attention to factors that were consistent predictors of future gambling problems, including a past history of gambling problems and intensity of gambling involvement. Key social determinants were also identified, such as the influence of family and friends, geographic proximity to electronic gaming machine venues, and mental health issues (Abbott et al. 2018). For instance, this expanding view of gambling research towards population impacts has helped fuel the production of evidence on the social costs of gambling (e.g., crime, socio-economic inequality, divorce) and comorbid health issues (e.g., mental illness, substance use, suicide) (Williams and Rehm 2011; Martin et al. 2014; Ronzitti et al. 2017; Price 2020).

These prospective studies have advanced the epidemiologic evidence base supporting a public health approach. Notably, the Canadian and the Massachusetts studies also included the Problem and Pathological Gambling Measure (PPGM) (Williams and Volberg 2010). This tool is unique in that it is designed to capture both risky gambling behaviours and harms. The dimensions of harm included are financial, mental health, physical health, and interpersonal relationships. The PPGM also incorporates work, school, and legal aspects of harm and asks about the impact they may have on an individual’s broader social network.

In the UK, the 2004 report on risk factors of problem gambling for the Responsibility in Gambling Trust elevated the discussion beyond the medical model. Key factors encompassed the *agent* (i.e., gambling exposure and availability), the *host* (i.e., personal characteristics and experiences that may make someone more or less likely to develop gambling problems), and *the environment* (i.e., the social, cultural, and physical setting within which gambling takes place) (Abbott et al. 2004). Consideration was also given to interactions between the domains in relation to the development of problems and harms as well as policy addressing harms from gambling.

A year later, in 2005, Gambling Research Australia produced a report assessing the existing body of evidence to define problem gambling and gambling-related harm. It also evaluated the merits of gambling screens and raised measurement issues (Neal et al. 2005).

By the end of the decade, it was clear that a movement towards population health frameworks and models informing researchers and studies was accelerating. It also brought to the forefront questions concerning definitions of gambling-related harm and new measurement techniques that would allow gambling-related harm to be assessed and monitored relative to public health practice and policy initiatives.

### The third decade: framework development

Public health research on gambling harms expanded in the 2010s as researchers grappled with conceptualization and measurement issues. In a bibliometric analysis of peer-reviewed gambling research published between 2008 and 2017 in three countries (Australia, New Zealand, and Canada), the number of harm-focused publications increased from 6.1% in 2008 to 23.5% of publications in 2017 (Baxter et al. 2019). New Zealand had the highest percentage of harm-focused gambling research publications during the 10-year period, followed closely by Australia. Canada lagged far behind, likely due to policy and research funding goals having more limited alignment with public health strategy for harm prevention compared to the other two countries. Overall, the increase in gambling harms research has led to new frameworks and the advancing understanding of gambling harm mechanics and impact. At the same time, there has been a movement towards not accepting research funding from industry to study gambling and related harms, consistent with research funding practices for other health issues such as tobacco and alcohol abuse (van Schalkwyk et al. 2019).

At the beginning of 2010, the Productivity Commission of the Australian government produced a detailed, two-volume report simply entitled *Gambling* (Productivity Commission 2010). In it, three models of gambling policy were presented: public health, consumer focus, and medical. The reported models provided clear distinctions in terms of population coverage, key goals, conceptual focus, policy tools, responsible departments, and key decision-makers (50, Fig. 3.3). The report laid the groundwork for evidence-based policy that would ideally include “measurement of environmental and individual risk factors, causality, and the incidence and prevalence of harmful outcomes” (50, Sect. 3.16).

**Fig. 1:**
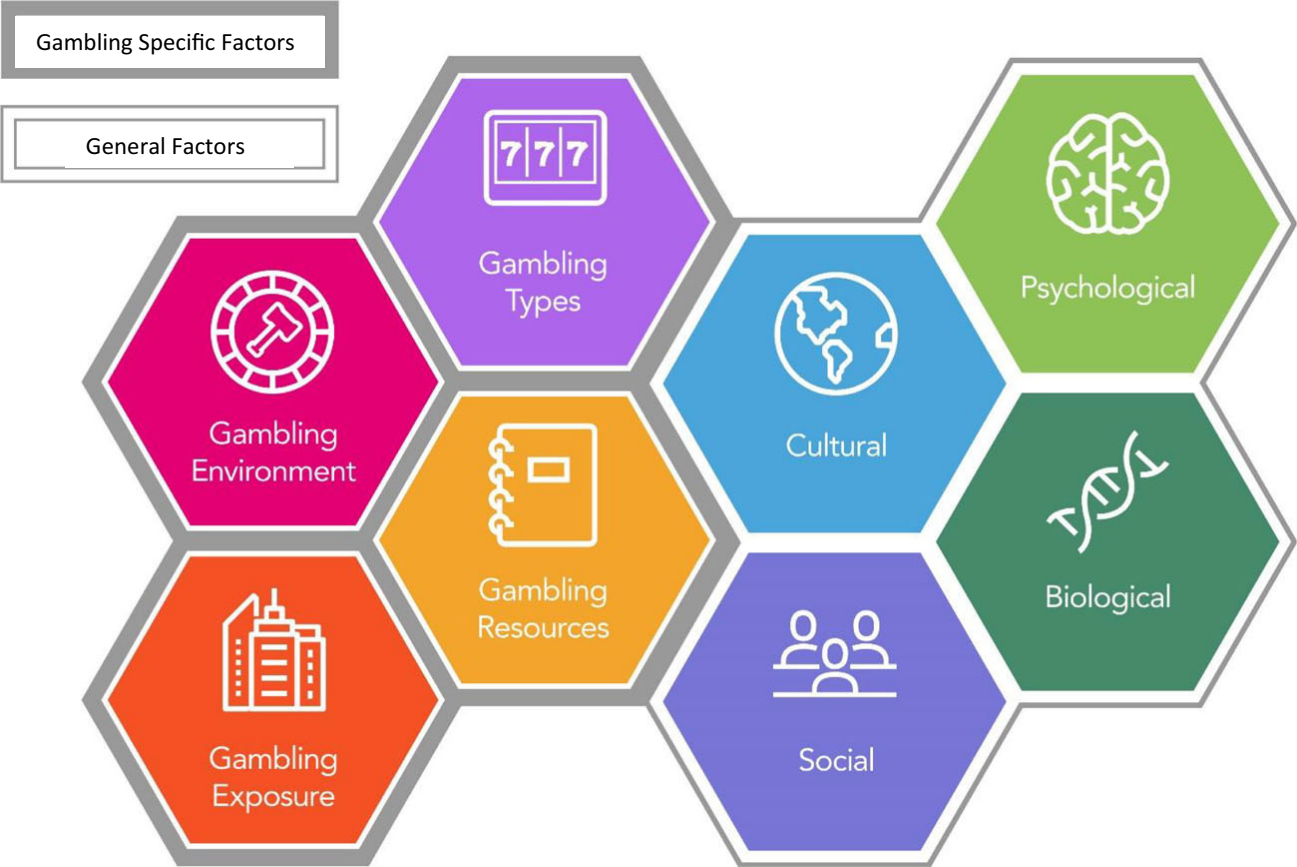
The Conceptual Framework of Harmful Gambling. Source: Abbott, M., Binde, P., Clark, L., Hodgins, D., Johnson, M. A., Manitowabi, D., . . . Williams, R. (2018). *Conceptual framework of harmful gambling: An international collaboration, third edition*. Retrieved from Guelph, CA: 10.33684/CFHG3.en, p. 6

Shortly thereafter, work began on the *Conceptual Framework of Harmful Gambling*, with the first edition published in 2013 (Abbott et al. 2013). Recognizing the complexity of gambling-related harm, the intention of the framework was to provide a comprehensive, multidisciplinary, international view of harmful gambling. Co-authored by a panel of international experts, the framework outlines antecedents and factors associated with harmful gambling. Eight interrelated factors, including four that are gambling-specific (gambling environment, gambling exposure, gambling types, and gambling resources) and four general factors (cultural, social, psychological, and biological) were identified (see Fig. 1). By positioning gambling harm as the organizing principle, the model more easily integrated health promotion strategies and provided guidance for corporate, regulatory, and public policy responses to reduce the potential harm from gambling. It further situated gambling harms within both public health and addictions disciplines. The framework is regularly updated to include new evidence and address issues of concern, with the most recent edition having been published in 2018 (Abbott et al. 2018).

**Fig. 2:**
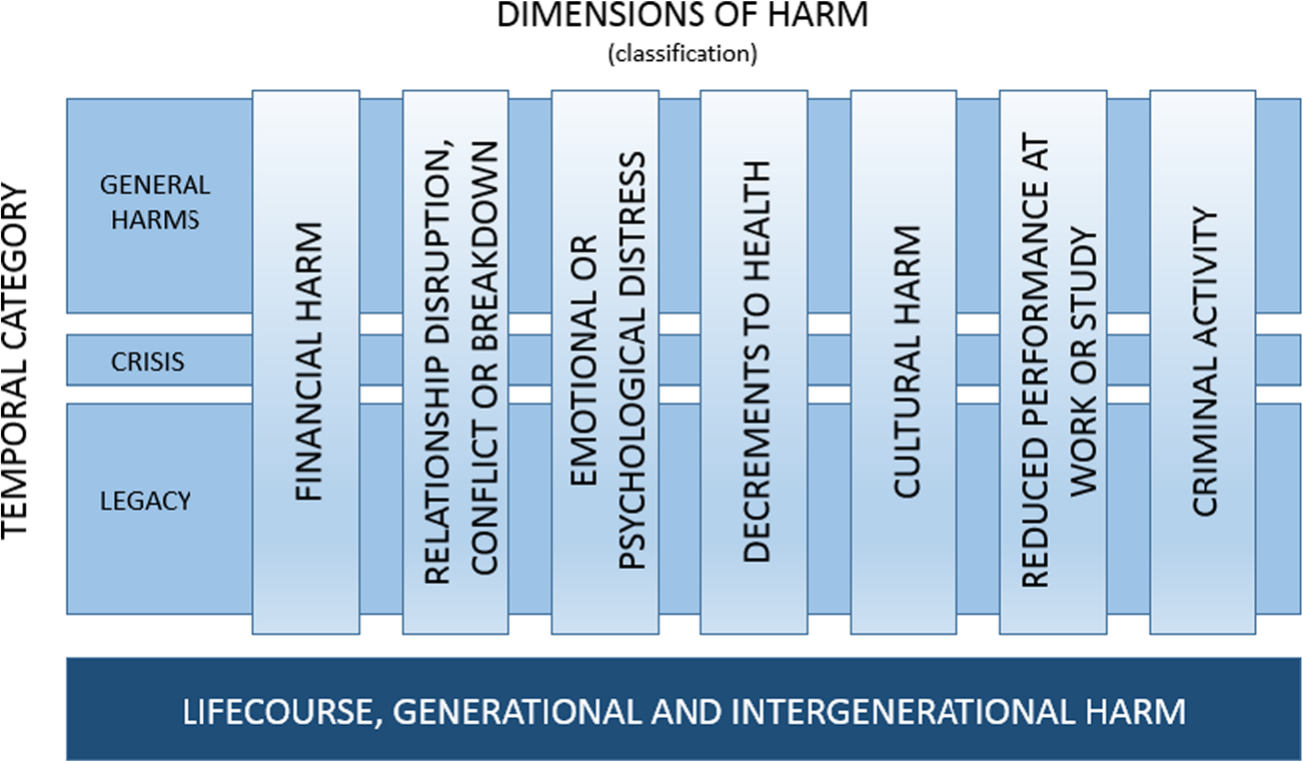
Conceptual Framework of Gambling-Related Harm. Source: Browne, M., Langham, E., Rawat, V., Greer, N., Li, E., Rose, J., . . . Goodwin, B. (2016). *Assessing gambling-related harm in Victoria: A public health perspective*. Retrieved from Victoria, Australia: https://www.responsiblegambling.vic.gov.au/information-and-resources/research/recent-research/assessing-gambling-related-harm-in-victoria-a-public-health-perspective, p. 40

A complementary study, the *Assessing Gambling-Related Harm in Victoria: A Public Health Perspective*, was developed by Australian researchers in 2016 (Browne et al. 2016). Funded by the Victorian Responsible Gambling Foundation, it followed a public health perspective and moved beyond antecedents and co-occurring conditions to identify and categorize harms that can result from gambling. Seven categories of harms are outlined, including financial, relationship disruption, emotional or psychological distress, cultural, health decrements, work or study performance, and criminal activity. Three temporal categories of general, crisis, and legacy harms address the stage of harm or level of severity. Underlying the dimensions and temporal categories are life-course, generational, and intergenerational harm, indicating the potential extension of gambling harm beyond the individual to affect opportunities and well-being for families and communities over time (see Fig. 2). Importantly, the study situates gambling at different risk levels relative to other health conditions in accordance with a burden-of-disease approach. Assessing the burden of disease is appropriate in measuring gambling harm because it can also reflect quality of life and be compared to other health issues using the same approach (Browne et al. 2017b). Data from the study provided a foundation for new measurement tools such as the Short Gambling Harm Screen (SGHS) (Browne et al. 2018) for use in population surveys. This is an important advancement, since commonly used prevalence measures, such as the Problem Gambling Severity Index (Ferris and Wynne 2001), were not intended to measure harm but are regularly used as a proxy. The report generated considerable discussion and has led to new research regarding the prevention paradox of public health for gambling (Browne and Rockloff 2018), along with critiques surrounding the burden-of-disease approach and measurements that could be interpreted as opportunity costs of gambling (e.g., see Delfabbro and King 2019).

**Fig. 3:**
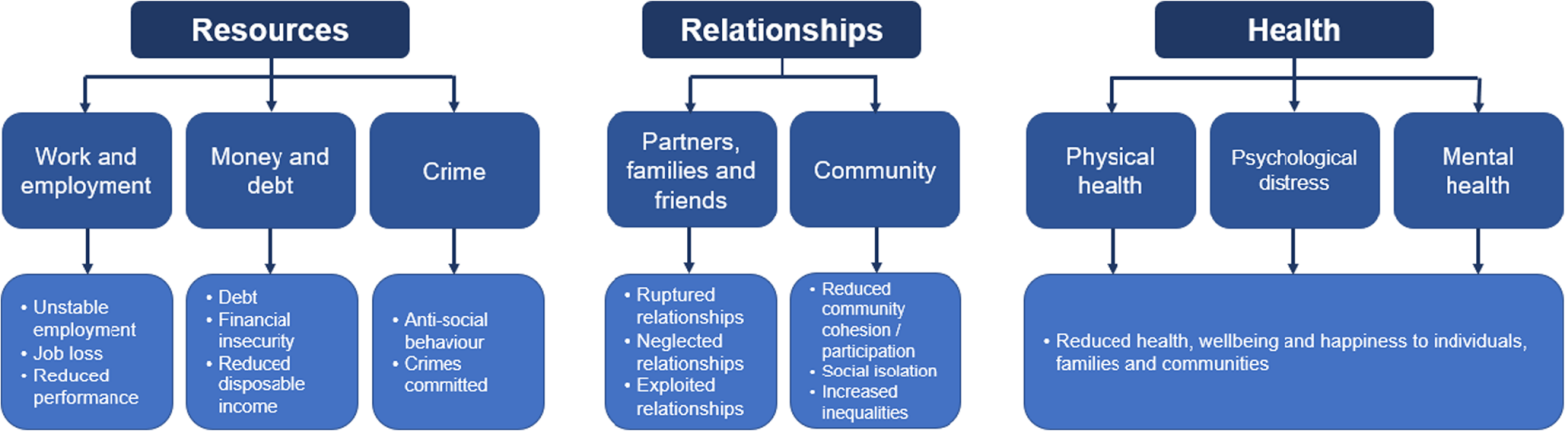
Source: Wardle, H., Reith, G., Best, D., McDaid, D., & Platt, S. (2018). *Measuring gambling-related harms: A framework for action*. Retrieved from Birmingham, UK: https://www.gamblingcommission.gov.uk/PDF/Measuring-gambling-related-harms.pdf. Adapted with permission from the UK Gambling Commission

In 2018, the Gambling Commission and GambleAware in Great Britain collaborated to produce a report, “*Measuring gambling-related harms: A framework for action”* (Wardle et al. 2018), that could inform strategic policy development on gambling harm reduction. Specifically, the report provided a working definition of gambling-related harms to situate it within a policy and regulatory action framework, and identified metrics and measures that could be used to estimate and monitor the social costs of harms. This is achieved by grouping gambling-related harms into three categories of *resources*, *relationships*, and *health*—each with two to three sub-factors—and using validated metrics to measure each resource category (see Fig. 3). Metrics were drawn from diverse sectors and measure indicators ranging from bankruptcy and/or debt relief orders to homelessness applications associated with gambling. The framework has since been adopted by government through the Gambling Commission’s 2019 *National Strategy to Reduce Harm from Gambling*, and now guides research and harm reduction initiatives in Great Britain (Gambling Commission 2019).

These three frameworks share a number of commonalities that align with a public health approach. Firstly, they all consider the effects of harm at multiple levels of impact, include harm across the complete spectrum of gambling behaviour, and recognize the complexity and diversity of contributing factors. The *Conceptual Framework of Harmful Gambling* (Abbott et al. 2018) was foundational in identifying and linking categories of factors that contribute to the risk and experiences of harmful gambling. The framework has been used by health professionals to guide conversations with patients and navigate challenges to treatment; applied in the development of research funding proposals to ensure that multiple domains of influence are considered; and served as a primer for students, research assistants, and other stakeholders to deepen their understanding of conditions that influence the risk of harm to gamblers and their significant others. The framework described in *Assessing Gambling-Related Harm in Victoria: A Public Health Perspective* (Browne et al. 2016) drew upon data from a comprehensive Australian research program to identify dimensions and temporal categories of harm from gambling that could be experienced by individuals, significant others, and communities. Examples of harms relevant to each level are presented in a taxonomy of harms (Langham et al. 2016), which formed the foundation of new harms measurement instruments. Another public health benefit is that as part of the research program, a burden-of-disease model was developed to position gambling-related harm relative to harm experienced from other health conditions. *The Framework for Action* (Wardle et al. 2018) was developed in Great Britain with policy advancement and the potential to measure social costs of gambling harm in mind. Recognizing that social costs are not always easy to identify, it nevertheless offers metrics for measuring harm using diverse databases from multiple sectors. Although the framework is specific to policy in Great Britain, many of the metrics, such as reduced credit scores, loss of trust between family members, or the experience of depression, are relevant across jurisdictions and could be adapted to public health programs in different settings.

Although other frameworks have been advanced during this decade (e.g., see Finnish researchers Latvala et al.’s (2019) Public Health Impacts of Gambling Model), they have not yet been as influential in shaping research and policy as those described above. With the expansion of new evidence-informed frameworks and models, the next logical step is to operationalize public health approaches to gambling harm, taking into consideration the inherent challenges and existing public health structures.

### Operationalizing a public health approach to gambling harm

Operationalizing a more comprehensive and coordinated approach to addressing gambling harm requires assessing how current efforts fit into the dominant service structures of public health systems. By examining where harm minimization efforts conform to the *essential functions* of public health systems, outlined below, we can better understand current public health capacity, areas for development, and potential barriers and challenges. The examples below provide illustrations of conformity and are not meant to be exhaustive or comprehensive.

#### Health promotion

Health promotion is a public health strategy that aims to improve the health of individuals by persuading them to adopt healthier lifestyles (Glouberman and Millar 2003). The most common tactics used in health promotion involve the provision of information and incentives, such as health education, social marketing, and program subsidies. It is distinct from other public health strategies, such as health protection, in that it targets individuals and not populations and is voluntary, based upon informed choice and not mandatory requirements.

*Responsible gambling* is a field of research and program development akin to health promotion in many ways—it is also the dominant strategy used in many jurisdictions to prevent gambling harms. In the early 1990s, gambling expansion in jurisdictions such as Canada saw the emergence of serious conflicts between health service providers, community advocacy groups, industry, and governments over the growing issue of gambling harm (Campbell and Smith 2003). Against this backdrop, many of these actors began engaging under the rubric of responsible gambling to come up with research, policy, and practices that could help reduce risk and prevent harm. Today, responsible gambling strategies commonly include gaming staff training on key topics (e.g., gambling risks, safer play practices, and assisting players with support service referrals) (Hing et al. 2018). The development and dissemination of print and digital information on gambling myths, signs of risk and harm, safer play habits, and personalized play history are also commonly included within this strategy (Lemaire and Dechant 2010; Mouneyrac et al. 2017; Parke et al. 2014). Interventions aligned with responsible gambling such as voluntary self-exclusion (e.g., self-banning) and pre-commitment (e.g., money and time limit-setting) can be viewed as program incentives supporting healthier lifestyle choices. In all of these cases, individuals are presented with information and a choice.

The attempt to purposefully align responsible gambling with public health approaches is probably best exemplified by the Reno Model (Blaszczynski et al. 2004). Published in 2004, it was one of the early strategic frameworks positioning gambling-related harm as a public health issue. The central focus of the model included the establishment of a global body of all key stakeholders to adopt principles of gambling harm reduction, collaborative planning, and scientific research and evaluation. The desire at the time was to develop clear metrics for gambling harm, guidelines for industry and gamblers to adopt, parameters for staff training, standards for advertising and health promotional messaging, and evaluation of policy and program impacts. While the Reno Model has seen adoption in many jurisdictions around the world, it—and responsible gambling more broadly—has faced critique mirroring that of health promotion in general (Abbott et al. 2018).

Like responsible gambling and the Reno Model, health promotion alone is considered to be a weak policy tool for improving population health (Bemelmans-Videc et al. 1998; Hancock and Smith 2017; Thompson et al. 2018). While responsible gambling initiatives such as those outlined in the Reno Model may be effective in raising awareness and educating the public on healthier gambling behaviours, the focus on personal responsibility for harm minimization has been widely critiqued and diminishes potential population impacts (Alexius 2017; Hancock and Smith 2017; Miller and Thomas 2018). This issue has also been addressed in relation to state responsibility, where the burden of harm is downloaded to the individual, rather than governments playing a more active role in harm prevention (Reith 2008; Miller et al. 2016; Reynolds et al. 2020). This is particularly relevant when competing advertising messages encourage consumption of potentially risky products. This was the case with tobacco and alcohol before greater emphasis was placed on structural interventions such as strict regulations, taxation, pricing controls, restrictions on availability, and marketing restrictions in various jurisdictions (De Beyer and Brigden 2004; Burton et al. 2017). For these reasons and others, several jurisdictions have chosen to use the term “safer gambling” rather than “responsible gambling,” since it is now widely recognized that factors beyond individual choice influence decisions to gamble.

#### Health protection

Health protection involves the assessment and control of potential health hazards in order to protect populations from harm. Legislation and regulation are the main policy tools supporting health protection, reflecting an emphasis on broader structural tools and requirements to change health behaviours. In public health, it traditionally focuses on environmental, occupational, and toxicological dimensions of health as well as food safety (World Health Organization 2018). Health protection also involves the management of communicable diseases or other threats to the public. In gambling, much like tobacco and alcohol, health protection may generally be reflected in policies or practices that aim to protect the public—often vulnerable populations, specifically—from undue and involuntary risk of harm.

Age restriction on legal gambling is a fairly typical form of policy supporting health protection. Some jurisdictions, such as Austria, have taken *player protection* much further and included requirements on operators to act if anyone is deemed unable to afford gambling based on income assessment or history of bankruptcy (Malischnig et al. 2018). Actions may involve a complete ban or limitations on frequency or time allowed to gamble. In Norway, the government requires that all players be registered for online or land-based gambling, and set mandatory loss limits that fall within global loss limits (Rossow and Hansen 2016; Auer et al. 2018). Evaluation of this health protection strategy involving over two million gamblers noted high approval ratings (79% among high-risk players, 91% among low-risk players) and adherence to limit reset periods (79% high-risk players, 95% low-risk players) without considerable participation in non-sanctioned gambling activities (Norsk Tipping 2016).

Areas where public health systems may already be positioned to leverage more support for gambling harm prevention relate to alcohol and other substance usage at land-based venues. Licensing requirements for dispensing alcohol at casinos, for instance, often note that service must be refused to anyone showing signs of intoxication and that staff must be trained appropriately to carry out this policy (Government of Ontario 1990). However, some public health organizations have pointed out that typical online staff training and enforcement can be weak (Centre for Addictions and Mental Health 2019). Gambling policy researchers have also noted that even if a player is refused alcohol service, they may be allowed to continue gambling (Hing 2004). The key issue in this case, warranting greater health protection efforts, is that alcohol consumption is a highly reliable risk factor for gambling problems and is associated with lower self-control and more impulsive behaviours (Lorains et al. 2011; Russell et al. 2019).

#### Disease prevention and harm minimization

Population-level prevention efforts focus on primary prevention of harm as well as associated risk factors before they have manifested, and secondary prevention to reduce or stop early signs of risk and harm (World Health Organization 2018). Examples of primary prevention programs in public health include efforts to address harm from alcohol abuse, unsafe sex, and poor nutrition (Yamada et al. 1999; Jaime and Lock 2009; Marsiglia et al. 2012). Like most examples of primary prevention in gambling, the focus has tended to be on adolescent education and delaying or preventing the onset of risky behaviour (Dickson et al. 2004; Ladouceur et al. 2013; Stewart and Wohl 2013).

Secondary prevention, focusing on those who may be at risk of gambling harm or have experienced early signs, is informed by effective screening tools and practices. In public health, screening programs can involve tests to assess morbidities such as cardiovascular disease, neurological diseases, and even mental health disorders. Screening for gambling risk and harm has often relied on self-assessment or staff-assisted questionnaires that have been developed and validated to detect behavioural risk factors. The Short Gambling Harm Screen (SGHS), the Problem Gambling Severity Index (PGSI), and the Problem and Pathological Gambling Measure (PPGM) represent some of the tools being used in the field. Clinical tools such as the Diagnostic and Statistical Manual of Mental Disorders, Fifth Edition (DSM-5) for disordered gambling are also used to assess risk of harm—the DSM-5 diagnostic tool is designed for individual clinical assessment, but has been applied to broader population-level health assessment such as prevalence studies. Most recently, the Canadian Centre on Substance Use and Addiction has been completing development of the world’s first empirically derived lower-risk gambling guidelines. These guidelines, based on 11 international gambling prevalence data sets, may help at-risk gamblers assess their behaviours as well as inform the public more broadly on safer, sustainable gambling practices (Canadian Centre on Substance Use and Addiction 2018). In principle, these guidelines will be similar to low-risk alcohol drinking guidelines.

Secondary prevention efforts in public health and in the gambling field also overlap with harm reduction approaches. Harm reduction approaches attempt to reduce morbidity and risky behaviours, such as substance use, when abstinence may not be feasible—although it remains a goal. High-profile examples of harm reduction in the public health field include the provision of supervised safe injection sites with clean needles for intravenous drug users who are at risk for overdose and infection from needle-sharing (e.g., HIV and hepatitis C infection). Programs like this have been found to be effective at reducing drug-related mortality and health care costs as well as increasing public safety (Wood et al. 2006). In the gambling field, voluntary self-exclusion, pre-commitment limit-setting, and counselling can be considered forms of preventive intervention for at-risk players—although they may also apply to primary prevention in some instances (Caillon et al. 2019; Nower and Blaszczynski 2010; Parke and Rigbye 2014; Rodda et al. 2013).

#### Population health assessment

Population health assessment involves the systematic collection and analysis of data, such as vital statistics on local health status, to identify health needs and key public health issues as well as to provide a basis for decision-making to plan prevention efforts (Ministry of Health and Long-Term Care 2018). Assessments can develop over years and provide broad levels of health information for long-term planning and policymaking. They may also include the regular monitoring of health system performance and analysis of measures to detect changes in the environment or health status of populations.

In the gambling field, the closest thing to population health assessment is gambling prevalence studies that measure self-reported gambling participation and screen for signs of risk. The *first decade* of gambling research and program development helped established this practice, as demonstrated in the 1990 New Zealand and 1991 Australian population surveys addressing the extent and severity of gambling-related problems (Abbott and Volberg 1992; Dickerson et al. 1996). Gambling prevalence studies have been instrumental in identifying key risk factors for gambling harm and helping to support preventive policy and program development (Toronto Public Health 2012; Volberg 1994; Wardle et al. 2011; Wood and Williams 2009). They have also informed the development of the Conceptual Framework of Harmful Gambling (Abbott et al. 2018) and Browne et al.’s (2016) framework of gambling harm. Despite these efforts, the production of gambling prevalence studies in most developed jurisdictions has been inconsistent in terms of regularity and comparability across (and even within) jurisdictions (Calado and Griffiths 2016). In addition, drug and mental health information systems that track health service utilization and screen for gambling-related harm can provide useful data to evaluate public need and service costs (Centre for Addiction and Mental Health 2017). There remains a notable gap in gambling research to guide public policy as well as patient management (The Lancet 2017). Ultimately, the incorporation of behavioural, social, and health service administration data has largely been lacking, resulting in limitations in targeted population interventions and service capacity improvement.

#### Health surveillance

Health surveillance involves the continuous, systematic collection, analysis, and interpretation of health data that is used for program development, implementation planning, and ongoing evaluation of public health practice (i.e., it focuses on program/practice/system performance outcomes). The provision of this type of information enables the detection and response to public health issues requiring immediate attention (BC Ministry of Health 2006). Health protection and emergency management can often be the beneficiaries of this type of information. For instance, sentinel surveillance systems distributed across health care units, such as hospitals, can collect high-quality data to detect the spread of highly infectious diseases (e.g., influenza, SARS, H1N1, COVID-19, etc.) and rapidly respond with preventive interventions. In addition, surveillance information can also support longer-term planning and system improvement.

It is not common that researchers or those working in gambling fields have ready access to current health surveillance data for monitoring gambling risk. One notable exception is in New Zealand, where the Kupe data explorer presents results the most current information from the Health and Lifestyles Surveys about gambling and other health concerns such as alcohol, tobacco, and mental health and well-being (Health Promotion Agency 2018). Many gambling operators, however, will collect and analyze data on key metrics such as rates of self-exclusion, those breaching self-exclusion agreements, red-flag incidents involving confrontations with gaming staff, large and increasing gambling losses by players, and other risk and harm metrics detectible through online gambling algorithms (Dragicevic et al. 2011; Schellinck and Schrans 2011; Ontario Lottery and Gaming Corporation 2016; Percy et al. 2016). Indeed, with the increased availability of online forms of gambling, the collection of behavioural data represents a unique opportunity to monitor player health for the purposes of targeting primary and secondary prevention efforts. Access to this data for public health gambling research and planning could prove extremely powerful. In addition, metrics identified in the Framework for Action (Wardle et al. 2018) would extend understandings, since they were chosen with a social determinants of health approach in mind.

## Discussion

Successful public health interventions take into account local settings. Gambling presents unique social, economic, political, regulatory, and technological contexts that vary by jurisdiction and deserve consideration in the design of public health approaches to harm prevention and minimization. For instance, the mix and design of some gambling products in a jurisdiction can influence patterns of consumption (Schull 2014). Two decades ago McMillen (2000) observed, and predicted, that global and digital technological advances related to online gambling would be a significant context and challenge for regulatory development, enforcement, and effective harm minimization strategies. In New Zealand, Adams and Rossen (2012) point out that institutional processes and policies can compromise a public health approach to gambling, and highlight the need to consider vested interests in design and development. In comparison with other public health issues (e.g., obesity, physical activity, alcohol policy, tobacco control, blood-borne viruses, and sexual health), Livingstone et al. (2019) conclude that public health interventions for gambling should rely on the best available evidence and must be plausible. With these points in mind, there are two foundational pillars to addressing gambling-related harm from a public health perspective: *making a case for gambling as a public health issue* and *the development of robust measures that align with a disease model* (Browne et al. 2017c).

While gambling harm may be perceived by public officials and policy actors as a *public health issue*, it is rare that it is institutionally operationalized and addressed by public health systems. One indicator of this phenomenon is the governance structure of legal gambling industries, which often feature ministries of finance, justice, or consumer affairs as their principal government authorities, and typically not ministries of health (Gambling Commission 2019; Kennedy 2019; Thompson 2019). These governing arrangements have the effect of excluding public health actors and departments from gambling-related policy development until gambling harm becomes viewed as a public crisis requiring immediate public health input. At present, New Zealand is the only country with a public health approach embedded in the Gambling Act. No others have followed, even though public health principles to approaching gambling were advanced almost two decades ago in other countries (Government of New Zealand 2003; Korn et al. 2003; Shaffer 2003) In recent years, the growing regulatory pressure and public interest surrounding gambling harm in Great Britain stands out as a notable case study (Davies 2017; Gambling Commission 2018; Kollewe 2019; British Broadcasting Corporation 2020). In response, a harm reduction strategy is now incorporated at the policy level in the Gambling Commission’s *National Strategy to Reduce Gambling Harms* (Gambling Commission 2019).

Solid epidemiologic evidence, in particular, is critical to fostering practical and policy-level changes in the field of gambling harm prevention and reduction. At the international level, Vladimir Poznyak, Coordinator of the World Health Organization Management of Substance Abuse program, points out that identifying significant impacts on health across the full spectrum of gambling behaviour is necessary to advance the public health agenda. This has also been noted in foundational documents across the decades including Korn and Shaffer’s public health framework (1999), the Australian Productivity Commission’s public health policy model (2010), and the more recent frameworks advanced in the Conceptual Framework of Gambling Harm (Abbott et al. 2018), the *Assessing Gambling-Related Harm in Victoria* study (Browne et al. 2016), and the Framework for Action in Great Britain (Wardle et al. 2018). Encouraging public health system involvement to leverage expertise and resources for preventive interventions—not just health promotional information—and data development and analysis represent key opportunities for the field’s advancement. Moreover, having consistent standards of harm minimization will enhance adoption, with international partnerships fulfilling a key role.

Population-level longitudinal studies have contributed to tracking risk factors, the etiology of problem gambling, and movement into and out of gambling risk categories over time. This practice contributes to population health assessment that serves the needs of evidence-informed policy and program development. Still, there are only six jurisdictions internationally in which these studies have taken place. More prospective longitudinal research with consistent, validated gambling harm measures across study jurisdictions is needed to clearly identify priority harms and population subgroups that are most vulnerable to negative outcomes. The inclusion of harm-specific measures such as the PPGM and the newer SGHS would build on the earlier Canadian and Massachusetts prospective studies that assessed gambling harm prevalence. In the interest of transparency, cross-jurisdictional support, and encouraging more research from a public health perspective, expanded longitudinal research would present an opportunity to share data widely among the gambling and public health research communities and help advance knowledge from multiple disciplinary perspectives. It would also help to fill the research gap identified by *The Lancet* (2017) where gambling and public health policy is concerned.

Beyond health assessment data, health surveillance data can enable monitoring and evaluation of immediate indicators of gambling risk and harm (e.g., the expansion of high-risk betting events or self-exclusions associated with new products). Although there is growing interest in integrated forms of gambling-related harm data showing gambling behaviour, treatment and counselling interactions (including third-party interactions), related bankruptcy filings, or credit counselling, much more work is needed to develop these systems. Again, the development of integrated health information systems is an area where public health systems have some expertise and knowledge to offer (Luić and Striber-Devaja 2006; Shah and Rogers 2019).

Another key challenge and opportunity relates to measurement tools. Proxy measures of harm often used in population surveys, such as the Problem Gambling Severity Index (PGSI) (Ferris and Wynne 2001), are useful for determining risk categories but have shortcomings in terms of measuring harm as an outcome (Langham et al. 2016). As mentioned earlier, the Problem and Pathological Gambling Measure (PPGM) (Williams and Volberg 2010) is an instrument used by some researchers to identify the incidence of specific gambling harms. An advantage of the PPGM is that it allows concurrent assessment of problem gambling and gambling-related harm, but a disadvantage is that it fails to capture the extent of harm being experienced by close relations. The more recent frameworks developed in Australia, including the harms taxonomy (Browne et al. 2016), and in Great Britain (Wardle et al. 2018) help to conceptualize what questions need to be asked, of whom, and in what context.

Researchers in Australia and New Zealand have done much to advance the public health agenda by using health-related quality of life measures, including burden of disease, to situate gambling risk levels relative to other health conditions (Adams and Rossen 2012; Browne et al. 2016, 2017a). Using data from the *Assessing Gambling-Related Harm in Victoria: A Public Health Perspective* study, the authors created a taxonomy that can be used as a checklist of harms at the individual, family, and community levels. This 72-item list of harms was subsequently shortened to a 10-item Short Gambling Harm Screen (SGHS). The abbreviated measure, featuring an exceptionally high correlation score (*r*_*s*_ = .94), is effective at capturing gambling-related harm at a level almost as high as the original 72-item checklist (Browne et al. 2018). As such, the SGHS presents a new opportunity for measurement that addresses gambling harm directly in a way that would have a relatively low participant burden.

Even so, the SGHS is not without critique. Some of the items in the SGHS may be perceived as trivial or inconsequential (e.g., being less able to spend money on other recreational activities), and perhaps represent an opportunity cost rather than a harm from gambling (Delfabbro and King 2019). Still, the high correlation between the SGHS and the 72-item checklist suggests that despite the potential of some items to be framed as an opportunity cost, they still capture perceived harm among people who gamble. Research using this technique consistently indicates that a larger proportion of the total burden of harm is associated with low-risk and moderate-risk gamblers (Browne et al. 2016, 2017c). Although the harms experienced by this group may not be as severe as those with problem gambling, by virtue of their greater numbers they are responsible for more harm at the population level. The high proportion of harms experienced by lower-risk categories aligns with the “prevention paradox”, which has been observed for other public health issues where prevention strategies are directed towards the small group at high-risk rather than the many others in low-risk categories (Browne et al. 2018; Delfabbro and King 2019; Rose 1981). The prevention paradox can be applied to gambling for the majority of harm categories, with some exceptions for rare and severe harms (Bourget et al. 2003; Canale et al. 2016; Browne and Rockloff 2018). In contrast, some critiques of the prevention paradox have argued that the misclassification of low-risk gamblers, scoring systems that under-represent the frequency of harms among high-risk groups, and conflation of behaviour as harm have not been adequately addressed (Delfabbro and King 2017, 2020).

The attribution of harms to gambling also continues to be an ongoing challenge. Gambling problems stem from complex and diverse social and economic factors (Abbott et al. 2018), and may be complicated by the high rate of comorbid health conditions. Up to 94% of people with gambling problems will have at least one co­occurring mental health or addiction disorder (Yakovenko and Hodgins 2018), with alcohol and nicotine dependence, depression, anxiety, and obsessive-compulsive disorder heavily represented (Crockford and el-Guebaly 1998; Lorains et al. 2011; Yakovenko and Hodgins 2018). It is likely that some gambling-related harms result from or are worsened by a combination of mental health and addiction disorders, yet fewer than one-fifth of people with gambling problems and psychiatric disorders are treated for these conditions concurrently (Browne and Rockloff 2018; Yakovenko and Hodgins 2018). Therefore, there is an opportunity for health professionals to screen for gambling problems when people present with mental health and substance use disorders. It may be that by treating the gambling problem, there will be a positive impact on comorbid conditions, or vice versa. Evidence further suggests that situating mental and behavioural disorders as an integral component of public health, from prevention through recovery, can lead to reduced health care costs overall and enhance recovery potential (Chen et al. 2018).

A pragmatic approach to measuring gambling-related harm could be to use measurement tools both within and outside the gambling sector. For example, Sweden tracks helpline call data as a rough measurement of harm experienced by gamblers and significant others. There have also been calls for financial institutions to monitor gambling transactions, as well as other sectors such as intimate partner violence services (where gambling is listed as a cause), bankruptcy courts, and coroners’ reports where gambling is indicated as a cause of suicide (as is done, for example, in Quebec, Canada (Bourget et al. 2003) and Hong Kong (Papaioannou et al. 2010)). Although it may be more challenging to draw upon diverse sectors and data sources to assess gambling-related harm, it also creates the opportunity to develop an integrated, or “syndemic”, network of stakeholders that could work towards a common public health goal to identify gambling problems at earlier stages and respond in a concerted manner to reduce or prevent harm from occurring (Nower and Caler 2018). This would involve building coalitions of key stakeholders and applying a public health lens to areas where people are most likely to suffer harm, and developing a regularly updated set of indicators drawn from credible and reliable sources. The importance of a base of foundational evidence is a key principle underlying a public health approach to gambling, as was noted by Shaffer in 2003 and is currently demonstrated by the Kupe data explorer supported by the New Zealand Health Promotion Agency.

## Limitations

This narrative review presents a few limitations that are important to note. Firstly, the process of literature collection and review was iterative and non-systematic, depending largely on the extensive experience and collaborative deliberation of the authors. As such, some relevant sources may have been overlooked, though the purpose of this paper has been to illustrate the general path of public health development in the gambling field. Similarly, this paper has attempted to overcome the considerable task of summarizing decades of research and development as well as placing this development within a public health model to determine relevant future paths for the field. Although the authors believe this has been successfully achieved, doing so within the scope of a single paper has required a sacrifice in how exhaustive this review can be. For instance, only English-language articles and documents were reviewed, even though it is acknowledged that valuable contributions to the field of gambling harm have been published in French, German, and other languages.

## Conclusion

Gambling is a globally prevalent activity engaged in by the majority of populations. It generates hundreds of billions of dollars annually for industry and governments (Casino.org 2016). However, for a large number of people and their close relations, gambling is associated with the experience of significant risk and harm (Calado and Griffiths 2016). As such, gambling harm can be considered a serious population and public health issue. Initial efforts to adopt a public health approach began in the 1990s following the New Zealand and Australia population surveys and have not yet been fully realized beyond New Zealand. To date, gambling harm has received most of its attention from downstream medical models, as evidenced by the contributions and emphasis of treatment and research professionals from the fields of psychology (Baxter et al. 2019). While this disciplinary orientation is vital to addressing risk and harms associated with gambling, upstream prevention at the population level requires further development.

Over the past few decades, momentum has been building to a public health approach that could address this need for upstream prevention of gambling-related harms. With recent and emerging developments in the areas of research, practice frameworks, and measurement tools, opportunities to integrate and operationalize public health approaches to gambling harm prevention appear more viable than ever. The early foundation of guiding principles, prospective studies, new frameworks, and advances in understanding the reach of gambling harm provide support to a public health approach.

When comparing developments in the gambling harm prevention and reduction field to the functions of modern public health systems, there is evidence of alignment and opportunities for further development and collaboration. For instance, safer gambling initiatives reflecting health promotion are well developed. Regulatory frameworks and preventive initiatives such as youth education and risk screening also reflect elements of health protection and harm prevention. To a lesser degree, health assessment and surveillance have featured contributions such as various prevalence studies and limited use of behavioural data from online operators. The Kupe data explorer (Health Promotion Agency 2018) provides an example of how data can be presented for rapid access to gambling and health information.

Moving forward, making the case that gambling harm is a public health issue and developing data to articulate the issue are the biggest challenges and opportunities for the field. Public health policymakers and practitioners can make meaningful contributions to the goal of gambling harm prevention by engaging on these two points and collaborating with those already working in the field. Areas where gambling harm intersects with other public health issues such as substance abuse, mental illness, poverty, and so on, can form a basis for developing integrated approaches to complex population health problems. A substantial evidence base that began in the 1990s on gambling and mental health comorbidities supports the need to take these factors into consideration. Prospective longitudinal studies have been pivotal in establishing a public health approach. They have demonstrated the movement between risk categories, and that prevention and intervention points could be addressed more thoroughly. New longitudinal studies with a consistent definition and measures of gambling harm are needed across jurisdictions.

Like tobacco and alcohol consumption, gambling is a complex policy issue with inherent cultural, social, and economic values that make addressing associated harms difficult to solve. While the public health approach may not provide a definitive solution to harm prevention, there are many indications it can help improve the current state of population health.
